# Early detection of mental illness for women suffering high-risk pregnancies: an explorative study on self-perceived burden during pregnancy and early postpartum depressive symptoms among Chinese women hospitalized with threatened preterm labour

**DOI:** 10.1186/s12888-020-02667-0

**Published:** 2020-05-20

**Authors:** Qianqian Ni, Guizhi Cheng, An Chen, Seppo Heinonen

**Affiliations:** 1 The First Affiliated Hospital of USTC, Division of Life Sciences and Medicine, University of Science and Technology of China, Hefei, 230001 Anhui China; 2 Institute of Healthcare Engineering, Management and Architecture (HEMA), Department of Industrial Engineering and Management, Aalto University, Maarintie 8, 02150 Espoo, Finland; 3 Department of Obstetrics and Gynaecology, Helsinki University Hospital and University of Helsinki, Haartmaninkatu 2, 00290 Helsinki, Finland

**Keywords:** Self-perceived burden, Early postpartum depressive disorders, Threatened preterm labour

## Abstract

**Background:**

The mental health of pregnant women, particularly those with elevated risks, has been an issue of global concern. Thus far, few studies have addressed the mental health of pregnant women with threatened preterm labour (TPL). This study investigated the prevalence of self-perceived burden (SPB) among Chinese women hospitalized due to TPL during pregnancy and early postpartum depressive disorders, exploring the effect of SPB and other potential risk factors on the early signs of postpartum depressive disorders.

**Methods:**

A self-reported survey was conducted in the obstetrics department of Anhui Provincial Hospital, China. Women hospitalized with TPL were approached 1 week after delivery. One hundred fifty women were recruited from January 2017 to December 2017. The Self-Perceived Burden Scale (SPBS) and Edinburgh Postnatal Depression Scale (EPDS) were the main measures. Descriptive statistics, Spearman correlations, and a multiple logistic regression were employed for data analysis.

**Results:**

SPB and early postpartum depressive disorders were commonly experienced by Chinese women hospitalized with TPL, and SPB was positively and significantly correlated with depressive symptoms. A multiple logistic regression analysis revealed that for the women hospitalized with TPL during pregnancy, the emotional aspect of SPB (OR = 1.42, 95% CI = 1.11–1.83, *p* = 0.006), age (OR = 1.14, 95% CI = 1.02–1.27, *p* = 0.023), occupation (OR = 3.48, 95% CI = 1.18–10.20, *p* = 0.023), the history of scarred uterus (OR = 7.96, 95% CI = 1.49–42.48, *p* = 0.015), the delivery mode of the present birth (OR = 6.19, 95% CI = 1.72–22.30, *p* = 0.005), and family support during pregnancy (OR = 0.60, 95% CI = 0.45–0.82, *p* = 0.001) were significant factors predicting early postpartum depressive symptoms.

**Conclusion:**

This study indicates that SPB and early postpartum depressive disorders are prevalent mental issues among Chinese women hospitalized with TPL, and that SPB, especially perceived emotional burden, is a strong predictor of early postpartum depressive disorders. Our study suggests the necessity of paying attention to mental health issues, e.g. SPB and postpartum depressive symptoms among hospitalized women with TPL, and providing appropriate interventions at the prenatal stage to prevent adverse consequences.

## Background

A high-risk pregnancy may involve traumatic events and introduce severe mental illness to a woman. A better understanding of women’s mental health in high-risk pregnancies is imperative and requires additional investigation [1, 2]. Threatened preterm labour (TPL) is one of most common indications of a high-risk pregnancy. Approximately 50% of women with TPL will present spontaneous preterm birth (sPTB) before 37 weeks, which is the leading cause of neonatal mortality and morbidity, causing over 70% of foetal deaths [3–7]. Not only is TPL a physical problem, but it also affects a pregnant woman’s psychological and emotional status [8]. However, as research in this area focuses on the causes and biological effects of TPL [9], few investigators have assessed the mental health of women with TPL before and after delivery. Among the limited number of studies, Papazisis et al. (2016) and Dagklis et al. (2018) estimate the prevalence of antenatal depression among pregnant women hospitalized in a high-risk pregnancy unit due to TPL and investigate its risk factors. With a slightly varied view, Carter et al. (2018) explore women’s experience of TPL [10–12]. In China, TPL accounts for approximately 15% of high-risk pregnancies [5]. China announced a universal two-child policy in October 2015, which was predicted to increase advanced maternal age-associated TPL in China and bring new challenges to obstetric care [13]. In this study, we investigate the prevalence and correlation of two mental issues—self-perceived burden (SPB) during pregnancy and postpartum depressive disorders—among women hospitalized with TPL at a tertiary care hospital in China.

SPB is defined as “empathic concern engendered from the impact on others of one’s illness and care needs, resulting in guilt, distress, feelings of responsibility, and diminished sense of self” [14]. SPB is understood as a multi-dimensional emotional construct arising from care recipients’ perceptions that they have become a burden to their caregivers [15, 16]. Equity theory [17] is an underlying theory of SPB, and according to equity theory, individuals try to maintain equity between the contributions that they bring to a social relation and the benefits that they receive from it, and if the balance is broken, emotional anxiety and depressive disorders may emerge. SPB has been found to be significantly correlated with decreased quality of life [18]. According to some recent studies, SPB will affect patients’ medical decisions and adherence [19]. Major research studies have shown that SPB is experienced by certain groups of patients, e.g., chronic disease patients [19], terminal cancer patients [16], and stroke victims [20] who experience physical symptoms (e.g., pain and physical weakness) and psychological difficulties (e.g., depression, anxiety, and decreased quality of life) [21]. To various degrees, these groups of patients rely on family members for living [20]. However, no attention has been paid to the SPB experienced by hospitalized pregnant women with TPL, who rely on others’ care and support and can suffer great physiological, psychological, social, and economic pressure. The Confucian concept of ethics combined with a family-oriented culture makes it exceptionally important to study the prevalence of SPB among Chinese women with TPL [22]. In China, young and middle-aged people are endowed with “pillar” roles in their families, and they are expected to shoulder the main family and social responsibilities, such as taking care of their parents and children and being breadwinners. Traditionally, women in China take the majority of family duties, including reproducing, raising children, and taking care of parents and parents-in-law. Relying on and being burdensome to family members, especially parents and parents-in-law, may lead women to perceive shame, significant guilt, and psychological burden. It has been demonstrated that in China, female patients generally tend to have a higher SPB than male patients [19]. Although China has witnessed revolutionary events and tremendous changes in the laws and policies related to marriage and family to protect and empower women, mothers still take the main responsibility in regard to parenting and shouldering household chores [23]. Themes such as hard work, sacrifice, and care [24] are entrenched in the motherhood of traditional Chinese families. Once mothers lose their confidence and ability to handle childbearing and parenting tasks, they may easily experience negative feelings, such as self-accusation and depression. Therefore, researchers and practitioners in women’s mental health should pay special attention to female patients hospitalized with high-risk pregnancies in countries with a family-oriented culture, like China.

Postpartum depressive disorders are among the most frequent psychiatric manifestations observed in women after childbirth [25], with various symptoms and severity in the spectrum from postpartum baby blues (depressed mood that occurs immediately after childbirth and that usually goes away in 3 to 5 days, with a prevalence of 30–75% [26]) to postpartum depression (PPD, a serious mental illness that involves a complex mix of physical, emotional, and behavioural changes happening to puerpera and that does not go away for longer than 2 weeks [27, 28], affecting 10–20% of mothers worldwide [25, 29]). There is a global consensus that postpartum depressive disorders exert negative short- and long-term effects on the establishment of motherhood, child development, and family wellbeing [30–33] and may lead to tragic consequences. For instance, PPD has been found to be a putative cause of disastrous behavioural tendencies, such as suicidal intent [34]. A substantial body of research has reported possible risk factors, e.g., socio-demographic factors, obstetric factors, the course of pregnancy and social support, for the development of postpartum depressive disorders [35–39]. Currently, improving the screening and diagnosis processes for postpartum mental illness, especially the provision of early detection and prevention of postpartum depressive disorders, has been an important task for all health professionals working with women during pregnancy and childbirth [40]. Some studies [41–43] have implied that it is important to identify women with depressed mood in the immediate postpartum period because it may increase their risk for developing major mental diseases at the later postpartum stage. Thus far, however, little attention has been paid to early postpartum depressive disorders among women who have experienced high-risk pregnancies, e.g., those who are hospitalized with TPL, likely suffer great physiological, psychological, social, emotional, and economic pressure during pregnancy, and require special care to recover from complications after delivery. Dagklis et al. (2018) indicate that thoughts of termination increase the risk of depression [11].

This study aims to investigate the prevalence of SPB during pregnancy and early postpartum depressive symptoms (1 week after delivery) among women hospitalized with TPL. We also aim to explore the effect of SPB and other potential risk factors on early signs of postpartum depressive disorders. This research can provide new theoretical and practical insights into women’s mental health during pregnancy and childbirth, and in particular, it can contribute to the early detection of postpartum depressive disorders for women with high-risk pregnancies. The results of this study will enrich the insights into women’s mental health in the course of pregnancy and childbirth, and assist obstetric professionals in designing and developing patient-centred health care services and improve clinical practices to effectively support pregnant women with high-risk pregnancies.

## Methods

### Design

This is an explorative study approved by the ethical committee of the First Affiliated Hospital of University of Science and Technology of China (USTC) (2019-P-013). We used a retrospective design to explore the prevalence of SPB and early postpartum depressive symptoms and their relationship. Women were asked to recall their affective and psychological status after being hospitalized with TPL and to assess their mood after delivery. All participants were informed regarding the aims, contents, and procedures of the study. A self-reported survey questionnaire was distributed between January 2017 and December 2017 in the obstetric department of the First Affiliated Hospital of USTC, that is, Anhui Provincial Hospital, a tertiary care hospital.

### Participants, recruitment process and data collection procedures

Based on previous estimates of the prevalence of postpartum depressive disorders among women [44] and considering women diagnosed with TPL and hospitalized, we assumed the prevalence of early postpartum depressive disorders in our study to be 75%. Using a formula and pre-defined parameters (*N* = 400Q/P, Q = 1-P, P = the prevalence of postpartum depressive disorders) [26] to calculate the sample size of our counting data, we needed a minimal sample size of 142.

The inclusion criteria for participants were as follows: pregnant women (1) diagnosed with TPL (Diagnostic criteria in Anhui Provincial Hospital: irregular contractions appearing before 37 weeks of pregnancy, with a small amount of vaginal bleeding, lower abdominal bulge, other preterm clinical manifestations, and dilatation of the uterine mouth < 2 cm [45]); (2) over 18 years of age; (3) without other severe neurological diseases or severe psychiatric disorders; (4) having adequate cognitive ability to provide credible information; (5) hospitalized with TPL and requiring special medical care to recover from complications until delivery; and (6) having at least one informal caregiver who could be a family member or friend and provided unpaid care.

**Fig. 1:**
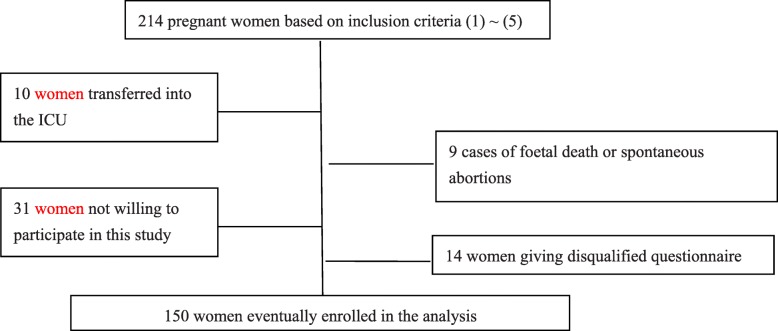
Flow chart of study recruitment process. Figure 1 Legends: In total, there were 214 pregnant women diagnosed with TPL and receiving prenatal care at the hospital. Among these eligible women, we had 9 cases of foetal death or spontaneous abortions (gestational age < 28 weeks [46]), 10 women were transferred to the intensive care unit during the study, 31 women were unwilling to participate in the study after delivery, and 14 women did not provide valid answers (e.g., all the answers were the same, or missing data constituted over 20% of the total items); these were excluded. As a result, a total of 150 pregnant women were included in the study. The flow chart of recruitment process is displayed in Fig.1

All women admitted to the obstetric department of the hospital were consecutively assessed for this study. In total, there were 214 pregnant women diagnosed with TPL and receiving prenatal care at the hospital. Among these eligible women, we had 9 cases of foetal death or spontaneous abortions (gestational age < 28 weeks [46]), 10 women were transferred to the intensive care unit during the study, 31 women were unwilling to participate in the study after delivery, and 14 women did not provide valid answers (e.g., all the answers were the same, or missing data constituted over 20% of the total items); these were excluded. As a result, a total of 150 pregnant women were included in the study. Statistically the actual sample size was adequate, since it was larger than the estimated sample size (142). The flow chart of recruitment process is displayed in Fig.1.

Women were approached 1 week after delivery by two trained senior nurses. In the hospital where we conducted this study in 2017, it was common for women to stay in hospital for recovery for at least 1 week after delivery, even with a normal delivery. Due to physical discomfort during pregnancy or for other reasons, women were not interested in participating in the survey before giving birth. After delivery, the puerpera were encouraged to recall their experiences of being hospitalized with TPL, and they reported their social, psychological and emotional status during their days in the hospital before and after delivery. The women were informed regarding the purpose and contents of the study and asked whether they would be willing to participate and fill out the questionnaire. The potential participants were also advised that the survey was voluntary, that their responses would be kept confidential, and that they could withdraw from the study at any time. The questionnaires were anonymous but stamped with an ID number. The questionnaires took approximately 20–30 min to complete. To facilitate their freedom to express their true feelings, the women were approached in the absence of their informal caregivers (e.g. husband, parents, and parents-in-law). In those rare cases in which women had difficulty understanding the questions or were unable to complete the questionnaire by themselves due to low literacy, the ward nurses read and explained the questions to them, the women responded to the questions, and the nurses then helped write the responses on the paper questionnaires. After each questionnaire was submitted, the nurses assessed whether all questions had been answered. If there were some unanswered questions, the women were kindly asked whether they had any confusion or issues regarding the questions, and nurses encouraged them to complete the unanswered items.

### Main measures

#### Self-perceived burden scale (SPBS)

The Self-Perceived Burden Scale (SPBS) was used to measure women’s self-perceived burden during pregnancy when hospitalized with TPL. Cousineau et al. [47] first proposed a conceptual framework of SPB and developed a 25-item scale SPBS 18.0. Subsequently, an abbreviated version of the SPBS consisting of 10 items was developed. In this study, SPB was measured with a validated, abbreviated Chinese version of the SPBS [48] that contains three dimensions (physical, emotional, and economic aspects) and 10 items. It uses a 5-point Likert scale, and each score is summed to create a score ranging from 0 (no burden) to 50 (maximal burden), with higher scores indicating more severe SPB. Scoring above 20 points indicates a noteworthy level of SPB [48]. The Cronbach’s alpha of the SPBS in our study was 0.799.

#### Edinburgh postnatal depression scale (EPDS)

In this study, we used the Edinburgh Postnatal Depression Scale (EPDS) [49] to assess women’s depressive disorders immediately postpartum (1 week after delivery). Some studies have suggested that the EPDS is a simple and useful tool for detecting the early onset of postpartum depressive disorders and that its administration in the 1st week postpartum can help to predict maternal mood at later postpartum stages [41–43]. The self-administered, locally validated EPDS consists of 10 statements on common depressive symptoms and uses 0–3 Likert-type responses to reflect the severity of symptoms [49]. In our study, we applied the recommended cut-off score of 10 [43] (sensitivity, 82%; specificity, 86%) to evaluate the level of postpartum depressive symptoms. In this study, the Cronbach’s α of the EPDS was 0.918.

#### Other variables

The socio-demographic characteristics and clinical information of the women were obtained from electronic medical records. The information included women’s parity (unipara/multipara), age, occupation (unemployed/employed), education (their highest level achieved, whether senior high school or below/junior college, or bachelor’s/master’s degree/doctorate), residence (rural/urban), number of terminations, having a scarred uterus before current delivery (no/yes), the use of assisted reproductive technologies (ARTs) (no/yes), the length of hospital stay for TPL before delivery (days), premature delivery (no/yes), and the delivery mode of the present birth (natural labour/caesarean delivery). We used the Multidimensional Scale of Perceived Social Support (MSPSS) to measure women’s perceived social support when they were hospitalized with TPL during pregnancy [50]. The MSPSS is a 12-item self-report scale that assesses perceived social support from three groups, namely, family, friends, and significant others. For each group, the MSPSS contains four specific statements and entails a 7-point Likert scale ranging from 1 (very strongly disagree) to 7 (very strongly agree). Item scores were aggregated, and a summed score ranging from 12 to 84 was obtained, with higher scores indicating greater perceived social support. The Cronbach’s α for the overall scale was 0.808. Information regarding insurance and expenses during TPL hospitalization was also obtained. However, since birth insurance can be used only once per year, in our study, all pregnant women with TPL chose not to use birth insurance for their TPL hospitalization, and we did not include expenses in our analysis since we believed that the economic aspect of SPB can directly reflect the economic burden perceived by women.

### Statistical analyses

Descriptive statistics were used to quantitatively describe or summarize basic features of the sample in our study (e.g., means, SDs, frequencies). We examined the associations of SPB with depressive symptoms in early puerperal period (1 week) using Spearman correlations [51]. To explore the risk factors for early postpartum depressive disorders, we used a multiple logistic regression model [51] with a stepwise approach, including SPB, perceived social support, and all socio-demographic and clinical factors available in the study as potential factors. We conducted all statistical analyses using SPSS 22.0. The *p* value of 0.05 was set for significance in the analysis.

## Results

### Sample characteristics

Table 1 depicts the sample characteristics of the 150 pregnant women; the mean age of the respondents was 30.9 ± 5.9 years (mean ± SD), ranging from 19 to 48. The number of gestational weeks when women were admitted to hospital for TPL was between 19^+ 0^ W and 36^+ 4^ W. All participants were married.

**Table 1:** Socio-demographic characteristics and clinical information of participants (*N* = 150)

Characteristic	Number participants (N)	Percentage (%)
Parity		
Unipara	62	41.3
Multipara	88	58.7
Age (years)	30.9^a^	5.9^b^
Occupation		
Unemployed	70	46.7
Employed	80	53.3
Education		
Senior high school or below	80	53.3
Junior college or Bachelor degree	64	42.7
Master or doctorate	6	4.0
Residence		
Rural area	44	29.3
Urban	106	70.7
The history of scarred uterus		
No	102	68
Yes	48	32
Number of termination	0^c^	6^d^
Assisted reproductive technologies		
No	132	88
Yes	18	12
Delivery mode		
Natural labour	48	32
Caesarean delivery	102	68
Length of hospital stay (day)	23.12^a^	16.30^b^
Premature delivery		
No	26	17.3
Yes	124	82.7
Social support (MSPSS)		
Family support	24.73^a^	2.99^b^
Friend support	21.99^a^	4.03^b^
Significant other support	22.60^a^	3.65^b^

^a^ = Mean, ^b^ = Standard deviation;

^c^ = Max ^d^ = Min

### Prevalence of SPB and early postpartum depressive disorders and their association

In our study, the participants’ EPDS scores ranged from 0 to 19, with a mean score of 9.67 (SD = 4.39), and a majority of pregnant women (54.7%) had EPDS scores above 10, indicating that early postpartum depressive disorders did indeed exist among the Chinese women who were hospitalized with TPL. The participants’ SPB scores ranged from 13 to 43, with a mean score of 22.53 (SD = 7.06), and over half of the women (64%) had an SPBS score above 20, indicating that a majority of the women in our study hospitalized with TPL were suffered from SPB during pregnancy. Table 2 shows sample distribution regarding postpartum depressive symptoms and SPB. The EPDS score was positively and significantly correlated with the full SPBS score (r = 0.50, *p* <  0.001), the SPBS-physical aspect score (r = 0.47, *p* <  0.001), and the SPBS-emotional aspect score (r = 0.51, *p* <  0.001), but there was no significant correlation with the SPBS-economic aspect score (r = − 0.05, *p* = 0.546). Table 3 shows the associations of postpartum depressive symptoms with SPB and its three dimensions.

**Table 2:** Sample distribution regarding postpartum depressive symptoms and self-perceived burden (N = 150)

		SPBS	
		< 20	> = 20
EPDS	< 10	34	34
	> = 10	20	62

**Table 3:** Relationship between postpartum depressive symptoms and SPB (N = 150)

Variables	Correlation coefficient (r)	*p*
Depressive symptoms (EPDS)	1	1.000
Self-perceived burden (SPBS)		
Total scores	0.50	< 0.001**
Physical aspects	0.47	< 0.001**
Emotional aspects	0.51	< 0.001**
Economic aspects	−0.05	0.546

*p < 0.05; **p < 0.001

### Multiple logistic regression analysis

We conducted a multiple logistic regression to explore the potential factors (including SPB) that influence early postpartum depressive symptoms. We divided the participants into non-depressive (< 10) and depressive (≥10) groups according to their EPDS scores. We set the three SPB subscales (physical aspect, emotional aspect, and emotional aspect) as the independent variables and included parity, age, occupation, education, residence, a history of scarred uterus, the number of terminations, assisted reproductive technologies, the length of hospital stay, the delivery mode, premature delivery, and perceived social support (family support/friend support/significant other support) as control variables (Table 4). We set dichotomous variables and coded the variables as follows: “0” for the no-risk category and “1” for the risk category [52].

**Table 4:** Assignments of the variables in the multiple logistic regression of postpartum depressive symptoms among participants (N = 150)

Variables	Assignments
Postpartum depressive symptoms (Y_1_)	0 = Not depressive,1 = Depressive
Parity(X_1_)	0 = Nulliparous,1 = Multiparous
Age (X_2_)	Actual measured value
Occupation (X_3_)	0 = Employed,1 = Unemployed
Education (X_4_)	1 = Senior high school or below,2 = Junior college or Bachelor degree,3 = Master or doctorate
Residence(X_5_)	0 = Rural area,1 = Urban
The history of scarred uterus(X_6_)	0 = Yes,1 = No
Number of termination(X_7_)	Actual measured value
Assisted reproductive technologies(X_8_)	0 = No,1 = Yes
Length of hospital stay(X_9_)	Actual measured value
Delivery mode	0 = Natural labour,1 = Caesarean delivery
Premature delivery(X_10_)	0 = No,1 = Yes
Social support	
Family support(X_11_)	Actual measured value
Friend support(X_12_)	Actual measured value
Significant other support(X_13_)	Actual measured value
Self-perceived burden	
Physical aspects(X_14_)	Actual measured value
Emotional aspects(X_15_)	Actual measured value
Economic aspects(X_16_)	Actual measured value

Y_n_ = The dependent variable, X_n_ = The independent variables

The results, in Table 5, showed that the emotional aspect of SPB (OR = 1.42, 95% CI = 1.11–1.83, *p* = 0.006) was a significant risk factor for early postpartum depressive symptoms, which means that women who perceived a higher emotional burden during hospitalization with TPL were more likely to develop early postpartum depressive disorders. In addition, age (OR = 1.14, 95% CI = 1.02–1.27, *p* = 0.023), occupation (OR = 3.48, 95% CI = 1.18–10.20, *p* = 0.023), the history of scarred uterus (OR = 7.96, 95% CI = 1.49–42.48, *p* = 0.015), the delivery mode of the present birth (OR = 6.19, 95% CI = 1.72–22.30, *p* = 0.005), and family support for women hospitalized with TPL during pregnancy (OR = 0.60, 95% CI = 0.48–0.82, *p* = 0.001) were predictive of early postpartum depressive disorders. Women who were at a higher maternal age, were unemployed, had no evidence of a scarred uterus, had a caesarean delivery as the delivery mode for the present birth, and/or did not have enough support from family during pregnancy were more likely to suffer early postpartum depressive disorders.

**Table 5:** Exploring influential factors for postpartum depressive symptoms (N = 150)

Predictors	B	S.E.	Wald	*p*	OR	95%CI		collinearity diagnostics
						upper	Lower	*VIF*^*a*^
Parity	0.42	0.72	0.34	0.558	1.53	0.37	6.29	2.70
Age (years)	0.13	0.06	5.20	0.023*	1.14	1.02	1.27	1.85
Occupation	1.25	0.55	5.14	0.023*	3.48	1.18	10.20	1.48
Education	0.23	0.45	0.26	0.612	1.25	0.52	3.01	1.48
Residence	0.06	0.61	0.01	0.925	1.06	0.32	3.50	1.48
The history of scarred uterus	2.07	0.85	5.89	0.015*	7.96	1.49	42.48	2.53
Number of termination	0.68	0.39	3.08	0.079	1.97	0.92	4.18	1.71
Assisted reproductive technologies	0.36	0.82	0.19	0.662	1.43	0.29	7.17	1.64
Delivery mode	1.82	0.65	7.79	0.005*	6.19	1.72	22.30	1.49
Length of hospital stay (day)	0.00	0.02	0.06	0.812	1.00	0.97	1.03	1.74
Premature delivery	−0.68	0.68	0.99	0.32	0.51	0.13	1.93	1.31
Social support								
Family support	−0.51	0.15	10.78	0.001*	0.60	0.45	0.82	2.39
Friend support	− 0.09	0.10	0.79	0.373	0.92	0.76	1.11	2.38
Significant other support	0.13	0.11	1.23	0.267	1.13	0.91	1.42	2.72
Self-perceived burden								
Physical aspects	−0.09	0.18	0.23	0.629	0.92	0.64	1.31	3.63
Emotional aspects	0.35	0.13	7.51	0.006*	1.42	1.11	1.83	3.24
Economic aspects	−0.47	0.29	2.66	0.103	0.63	0.36	1.10	1.29
Constant	4.42	3.62	1.49	0.222	83.15			

*p < 0.05; **p < 0.001

^a.^*VIF* Variance Inflation Factor

Our model explained between 40.1% (Cox and Snell R^2^) and 53.6% (Nagelkerke R^2^) of the likelihood of being screened positive for early postpartum depressive disorders. Since the results from variance inflation factor (VIF) analysis were between 1.31 and 3.63, we estimated that multicollinearity in the model was not strong.

## Discussion

Our findings indicate that women hospitalized with TPL in China commonly experience SPB. Chinese women hospitalized with TPL during pregnancy may feel that they are unable to contribute equally to a caregiving relationship due to the unforeseen situation and perceive themselves to be a burden to others (primarily family caregivers). We noticed that it is common for TPL women who suffer SPB to have early-onset postpartum depressive disorders. It is noteworthy that perceived emotional burden during pregnancy is a strong sign of postpartum depressive symptoms in women hospitalized with TPL. This finding scientifically responds to the on-going call for prenatally identifying women at risk for postpartum mental illness and preventing it as early as possible [53–55]. Our study enriches knowledge regarding the antenatal risk factors of early postpartum depressive disorders [44]. We suggest that assisting women hospitalized with high-risk pregnancies such as TPL in relieving SPB during pregnancy, especially reducing perceived emotional burden, could be an effective way to prevent them from suffering postpartum depressive disorders.

To the best of our knowledge, to date, this is the only study revealing that early postpartum depressive disorders were prevalent among women hospitalized due to TPL in the immediate postpartum period (54.7%), doubling the figure of 27.56% presented in one recent study depicting normal pregnant women’s depressive symptoms after 1 week of delivery [56]. Our findings deliver a message similar to that of Verdoux et al. (2002) [57], who concluded that women with obstetrical complications such as threatened abortion/preterm birth (seen as a severe life event for a pregnant woman during pregnancy) were more likely to present with severe depressive symptoms in the early postnatal period. This underlines the necessity of not only exploring the possible consequences of pregnancy complications for the physical health of both the baby and the mother but also being concerned with their continuous impacts on the mother’s mental health [57].

While the majority of studies have emphasized the importance of post-delivery family support in decreasing the risk of women experiencing depressive symptoms [39, 58], our results suggest that for women with high-risk pregnancies, obtaining enough family support during pregnancy (especially during the hospitalization period) is important in preventing them from developing postpartum depressive disorders. Thus, health professionals and/or social workers should help in formulating positive family relationships and encourage and advise women’s partners and other significant ones to give sufficient psychological and emotional support to women with high-risk pregnancies. In China, many studies on women’s health have confirmed that conflicts with their mothers-in-law constitute a risk factor for depressive symptoms [59]. While, based on the Confucian paradigm, a woman is expected to take care of, show respect for and obedience to her mother-in-law, the mother-in-law, in turn, is expected to play a key role in taking care of her daughter-in-law during pregnancy and childbirth. A previous study from China [37] reported that 48.8% of women had their mothers-in-law giving care after delivery. Undoubtedly, building a favourable relationship between women and their mothers-in-law is one of the keys to reducing the risk of postpartum depressive disorders.

We found that pregnant women with a higher maternal age were more likely to be depressed after delivery, which is in line with many other studies [38] [60], including a study by Ming Gao et al. [61]. It has been reported that pregnant women older than 30 years of age have a higher risk of PPD than other pregnant women. Similar to recent studies [62–65], our study showed that employment status was an influential factor for early postpartum depressive disorders. Women who are unemployed have an increased risk of early postpartum depressive disorders, probably due to perceived social exclusion and economic reliance on partners. We also observed that the mode of delivery was an important factor affecting the occurrence of depressive symptoms, as women who have caesarean deliveries demonstrated a greater risk of early postpartum depressive disorders. This result is in agreement with a plethora of other studies [66, 67]. The stress of a caesarean delivery and the complications that occur during and after a caesarean delivery are likely to induce the occurrence of depressive symptoms [68]. Our finding that women without a scarred uterus have a higher risk of suffering early postpartum depressive disorders was unexpected and certainly warrants further research. The previous findings [69–71] indicating that premature delivery is a strong risk factor for women developing early postpartum depressive disorders were not supported by the present study. This may be because for pregnant women hospitalized with TPL, premature delivery was already expected, and these women were prepared for unpleasant results. This also alerts us to the possible psychological consequences of experiencing TPL—a stressful life event—which need to be recognized and addressed regardless of whether the medical outcome of the pregnancy complication is favourable (i.e., when threatened abortion does not lead to premature birth) or not [57].

### Strengths and limitations of the study

To the best of our knowledge, this is the first study to reveal the prevalence of and association between SPB and postpartum depressive disorders among women with high-risk pregnancies, i.e., being hospitalized with TPL. This study provides new knowledge on the early detection of mental illness and provides novel insights into managing women’s mental health during pregnancy and childbirth, especially for high-risk pregnancies. We took some measures to increase the quality of this study and the credibility of the results, e.g., asking women to fill out the forms shortly after delivery but before discharge and examining multicollinearity among variables before regression analysis.

However, the present study has some limitations. First, because the data were collected via a self-reported survey with a retrospective design, the results of this study could be prone to memory bias and subjective self-assessment, which is a common issue in psychological research. To obtain a more accurate and confirmative conclusion, a prospective cohort study should be organized to observe the variation in and progression of women’s psychological and emotional status in the course of pregnancy and childbirth. Second, participants were recruited from a department of obstetrics at a single tertiary hospital and do not represent all hospitalized pregnant women with TPL in China or globally. It may therefore be problematic to apply the results of this study to other institutions or geographic regions. We expect to further this study by expanding the research context. Third, as the regression model we applied in this study explained between 40.1% (Cox and Snell R^2^) and 53.6% (Nagelkerke R^2^) of the likelihood of being screened positive for early postpartum depressive disorders, there are evidently other important risk factors that have not been included. Thus, in future studies, we will explore other factors that potentially underlie postpartum depressive disorders, including the personality characteristics of the pregnant women, knowledge of TPL, relationships with caregivers, the health condition of the caregiver, and so on. Last but not least, this exploratory study, investigating the mental health of women hospitalized with TPL, did not take women without TPL as comparing cases. A case control study will help to further develop this work.

## Conclusions

In our study, we identified SPB and postpartum depressive disorders as prevalent and noteworthy issues among women hospitalized with TPL, and we revealed that women’s perceived emotional burden during TPL hospitalization was a predictive factor of depressive disorders in the early postnatal period. By exploring the influences of demographic and clinical factors on early postpartum depressive symptoms, our study showed that maternal age, employment, a scarred uterus, and the delivery mode of the present birth are predictive of early postpartum depressive disorders. Our study suggests the necessity of paying attention to mental issues, e.g. SPB and early postpartum depressive, among hospitalized women with TPL and of providing appropriate interventions at the prenatal stage to prevent adverse consequences.

## Data Availability

The dataset generated and analysed for this study is not publicly available due to the restrictions claimed in the document of the research permission and ethical approval. But the data are available from the ethics committee of the First Affiliated Hospital Ethics Committee of USTC for researchers who meet the criteria for access to confidential data. To request access to the data, please contact the ethics committee of the First Affiliated Hospital Ethics Committee of USTC or the main researcher Qianqian Ni.

## Abbreviations

SPB: Self-Perceived Burden
PPD: Postpartum Depression
SPBS: Self-Perceived Burden Scale
EPDS: Edinburgh Postnatal Depression Scale
MSPSS: Multidimensional Scale of Perceived Social Support
VIF: Variance Inflation Factor
